# Minimally Invasive Approach of a Retrocaval Ureter

**DOI:** 10.1155/2016/3591832

**Published:** 2016-08-21

**Authors:** Nuno Fidalgo, Hugo Pinheiro, Frederico Ferronha, Jorge Morales, Luís Campos Pinheiro

**Affiliations:** ^1^Departamento de Urologia, Hospital das Forças Armadas, Azinhaga dos Ulmeiros, 1649-020 Lisboa, Portugal; ^2^Serviço de Urologia, Centro Hospitalar de Lisboa Central, EPE, Rua José António Serrano, 1150-199 Lisboa, Portugal

## Abstract

The retrocaval ureter is a rare congenital entity, classically managed with open pyeloplasty techniques. The experience obtained with the laparoscopic approach of other more frequent causes of ureteropelvic junction (UPJ) obstruction has opened the method for the minimally invasive approach of the retrocaval ureter. In our paper, we describe a clinical case of a right retrocaval ureter managed successfully with laparoscopic dismembered pyeloplasty. The main standpoints of the procedure are described. Our results were similar to others published by other urologic centers, which demonstrates the safety and feasibility of the procedure for this condition.

## 1. Introduction

The retrocaval ureter is a rare congenital entity that causes external compression of the proximal ureter and usually becomes symptomatic in the third or fourth decade of life [1]. For the treatment of this condition, classical open pyeloplasty techniques had been the gold standard for many years. In 1994, Baba et al. were the first to report a successful laparoscopic pyeloplasty for a retrocaval ureter [2]. Over the time, other reports were presented with good results in less time. Current evidence supports the laparoscopic approach as fist-line treatment for this condition.

## 2. Case Presentation 

**Figure 1:**
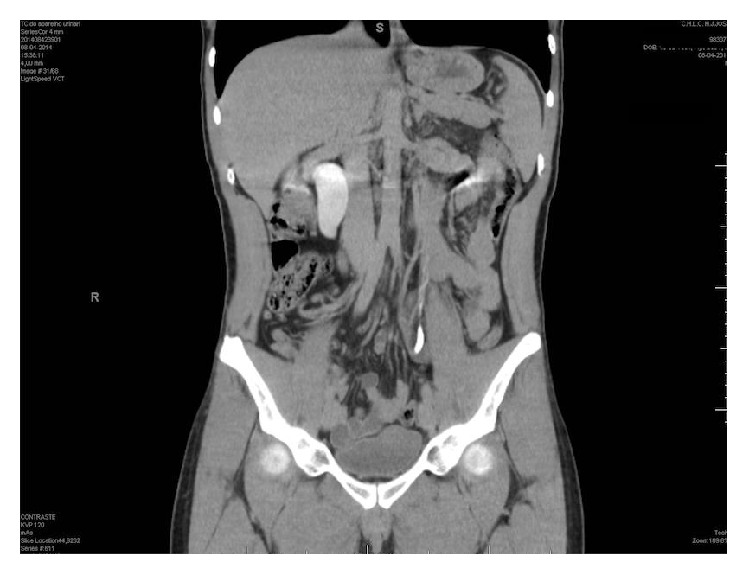
CT scan image showing the “reverse J” deformity.

We present the clinical case of a 35-year-old male with 12-month history of intermittent right flank pain. Physical examination and laboratorial investigation tests were unremarkable. Computed tomography (CT) scan after contrast infusion showed right hydroureteronephrosis, with the classical “reverse J” or “fishhook” deformity suggesting the presence of a retrocaval ureter (Figure 1) [3]. The mercaptoacetyltriglycine (MAG-III) renal scan showed right-side obstruction with a split function of 41.1% on the right kidney.

Therefore, the patient was proposed to undergo laparoscopic transperitoneal dismembered pyeloplasty.

**Figure 2:**
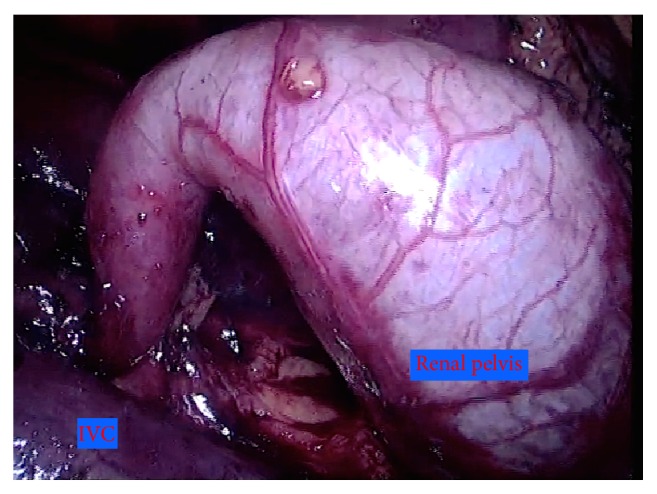
The renal pelvis after dissection.

The patient was placed in the left modified flank position at 45°, after induction of general anesthesia. We used a four-port approach with a 11 mm port to the right of the umbilicus, a 11 mm port half way between the first port and the right costal margin (in the midclavicular line), a 5 mm port in the midline (respecting the triangulation rule), and a 5 mm port in the right iliac fossa for suction device. The classic operative steps were performed for exposure: reflection of the ascending colon medially, identification of the ureter, and dissection of the right renal pelvis (Figure 2).

**Figure 3:**
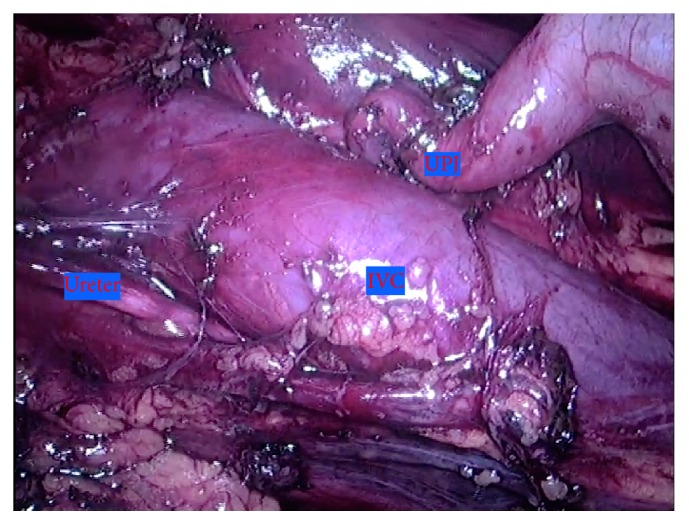
Renal pelvis, proximal ureter, and retrocaval portion dissected and mobilized.

**Figure 4:**
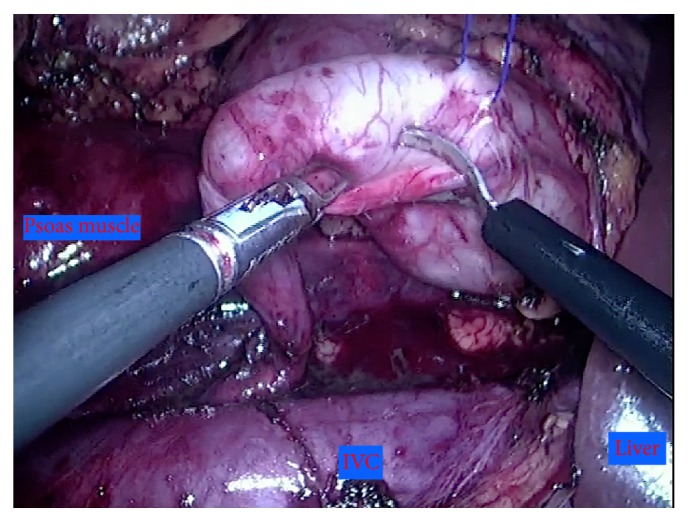
Preparing for pyeloplasty after suspension of the renal pelvis.

Careful dissection of the ureter was performed from the lateral border of the inferior vena cava (IVC) with the use of blunt dissection and bipolar device. Complete mobilization of the retrocaval portion of the ureter was achieved exposing its atretic and scarred portion (Figure 3). Then, we performed excision of the atretic and redundant portion and transposition of the ureter to an anterior position regarding the inferior vena cava. Previous to the reconstructive phase of the operation, we chose to suspend the renal pelvis to the anterior abdominal wall (passing a monofilament wire trough the renal pelvis and the abdominal wall with a straight needle), improving visualization and stabilization and dismissing the need for an “extra hand” (Figure 4).

**Figure 5:**
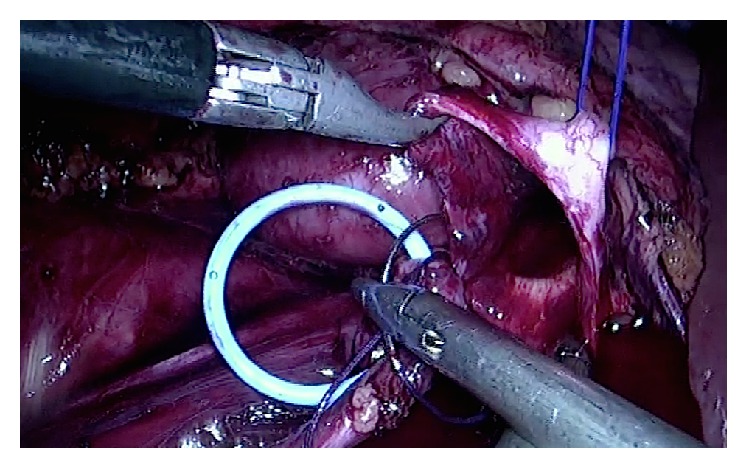
Starting the anastomosis, first on the posterior side with running suture.

**Figure 6:**
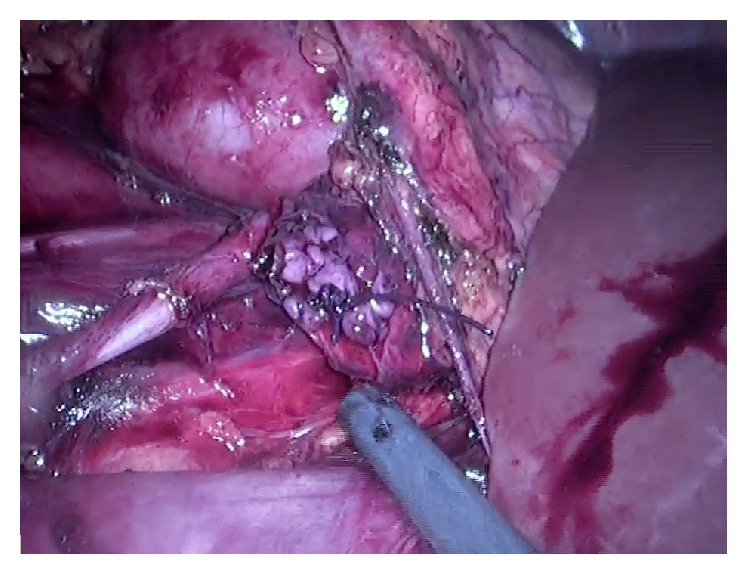
Final look of the anastomosis after inspection.

Classical steps for pyeloplasty were then performed: spatulation of the ureter, introduction of a 6 Ch 26 cm double-J stent in an antegrade fashion down the ureter into the bladder (passed along a 0.035-inch glidewire). The anastomosis was performed using 3-0 polyglactin sutures in a continuous, tension-free fashion (Figure 5). Care was taken to place de proximal curl of the stent in the renal pelvis. The anastomosis was finally inspected confirming water tightness (Figure 6). A closed suction drain was placed. Blood loss was minimal and total operative time was 170 minutes.

The postoperative course was uneventful. Closed suction drain was removed at 48 h. The patient was discharged at 72 h. We removed the double-J stent after 6 weeks in the office. Pathology processing of the excised portion of the ureter revealed signs of chronic inflammation and fibrosis. At 3-month postoperative consult, the patient presented symptom-free and a MAG-3 scan was performed showing no signs of obstruction. A postoperative CT urography was also performed at 3 months and showed normal contrast drainage and no sign of complications.

## 3. Discussion

The retrocaval ureter is a rare congenital entity that causes external compression of the proximal ureter and usually becomes symptomatic in the third or fourth decade of life. Hoechstetter first described it in 1893 emphasizing the anatomical basis of this condition [15]. However, the developing of this clinical entity is due to a vascular malformation, making the designation preureteric vena cava more embryologically accurate. Several theories tried to explain this condition. The one described by Shulman in 1997, which states the persistence of the subcardinal vein as IVC, seems to be the most accepted one [16]. Others suggest the persistence of the posterior cardinal veins developing the IVC. Regardless of the theory, we find that the failure of the supracardinal vein to persist as IVC is a common point [17].

The surgical treatment of the retrocaval ureter is indicated in the evidence of signs or symptoms of obstruction [4, 5, 18]. For the treatment of this condition, classical open pyeloplasty techniques had been the gold standard for many years [19]. The first successful open dismembered pyeloplasty, published by Anderson and Hynes in 1949, was performed on a retrocaval ureter [20]. In 1994, Baba et al. were the first to report a successful laparoscopic pyeloplasty for a retrocaval ureter, with a total operative time of 560 minutes. With time, the experience and the lessons learned with other laparoscopic procedures, especially when involving intracorporeal suturing techniques, opened way for the standardization of the laparoscopic approach for retrocaval ureter all over the world [6]. In fact, we see reports of different laparoscopic approaches (transperitoneal, retroperitoneal, and laparoscopic assisted with extracorporeal anastomosis and laparoendoscopic single-site surgery (LESS)), reflecting the experience of each urologic center in the field of Laparoscopy in Urology, applied in retrocaval ureter surgery [7–11] (Table 1).

In our case we chose to perform pyeloplasty, instead of simple ureteroureterostomy, because of two reasons. First, the ureter looked very redundant, and to perform ureteroureterostomy it would also be necessary to excise a large portion of healthy ureter in order to give the ureter a more anatomical and functional aspect. The second reason regards the experience with successful laparoscopic pyeloplasty in our department. Pyeloplasty seemed easier to perform and less likely to develop stricture since we reconstruct a larger caliber structure. We could also expect better blood supply once the anastomosis is performed more apically. These all made strong points in the technical choice.

We found no need to additional placement of ureteral stent before the procedure, like in laparoscopic pyeloplasty for other causes of obstruction.

From the technical standpoint, we also remarked some aspects that we find essential for the safety and reproducibility of this minimal invasive procedure: suspension of the renal pelvis to the abdominal wall prior to the anastomosis is an easy and quick step which improves visualization and stabilization and dismisses the need for an extra port placement.

As in other fields of urologic surgery, robotic surgery of the retrocaval ureter was also reported in the literature. It seems that both robotic assisted repair and pure laparoscopic repair offer the same advantages for retrocaval ureter surgery, offering quick recovery and good cosmetic results. Apart from the ergonomic ease for the surgeon and easier intracorporeal suturing provided by robotics, current evidence favours both approaches at the same level, as far as results are concerned [12–14].

## 4. Conclusions

Laparoscopic dismembered pyeloplasty is the standard of care for the treatment of ureteropelvic junction obstruction. When facing a retrocaval ureter, additional challenges emerge. Despite these challenges, it is possible to maintain the advantages of minimal invasive treatment: quick convalescence and few complaints with excellent functional outcome.

## Figures and Tables

**Table 1:** Case reports in the literature of surgical repair of retrocaval ureter.

Study	Cases	Approach	Procedure	Operative time (min)	Complications	Hospital stay (days)	Follow-up (months)
Baba et al. [2]	1	Transperitoneal	Pyeloplasty	560	None	Not reported	2
Mahmood et al. [4]	1	Open	Ureteroureterostomy	Not reported	None	Not reported	Not reported
Hyseni et al. [5]	1	Open	Ureteroureterostomy	Not reported	None	Not reported	3
Júnior et al. [6]	1	Transperitoneal	Pyeloureterostomy	210	None	4	6
Tobias-Machado et al. [7]	1	Retroperitoneal	Ureteroureterostomy, extracorporeal	130	None	2	3
Chung and Gill [8]	1	Transperitoneal	Pyeloplasty	180	None	2	6
Nagraj et al. [9]	1	Transperitoneal	Ureteroureterostomy	100	None	3	Not reported
Ricciardulli et al. [10]	27	Retroperitoneal	Ureteroureterostomy	131 (median)	4 cases	3.8 (median)	3, 6, and 12
Autorino et al. [11]	1	Transperitoneal LESS	Ureteroureterostomy	180	None	2	3
Hemal et al. [12]	1	Robotic	Pyelopyelostomy	Not reported	None	3	3
Nayak et al. [13]	5	Robotic	Pyelopyelostomy and ureteroureterostomy	92 (median)	None	2	13.5 (median)
Alkhudair et al. [14]	1	Robotic	Ureteroureterostomy	90	None	Not reported	3
